# Genomic Characteristics Distinguish Geographically Distributed *Dehalococcoidia*

**DOI:** 10.3389/fmicb.2020.546063

**Published:** 2020-09-08

**Authors:** Yi Yang, Yaozhi Zhang, Natalie L. Cápiro, Jun Yan

**Affiliations:** ^1^Key Laboratory of Pollution Ecology and Environmental Engineering, Institute of Applied Ecology, Chinese Academy of Sciences, Shenyang, China; ^2^University of Chinese Academy of Sciences, Beijing, China; ^3^Department of Civil and Environmental Engineering, Auburn University, Auburn, AL, United States

**Keywords:** *Dehalococcoidia*, genome comparison, evolution, time tree, reductive dehalogenase

## Abstract

*Dehalococcoidia* (*Dia*) class microorganisms are frequently found in various pristine and contaminated environments. Metagenome-assembled genomes (MAGs) and single-cell amplified genomes (SAGs) studies have substantially improved the understanding of *Dia* microbial ecology and evolution; however, an updated thorough investigation on the genomic and evolutionary characteristics of *Dia* microorganisms distributed in geographically distinct environments has not been implemented. In this study, we analyzed available genomic data to unravel *Dia* evolutionary and metabolic traits. Based on the phylogeny of 16S rRNA genes retrieved from sixty-seven genomes, *Dia* microorganisms can be categorized into three groups, the terrestrial cluster that contains all *Dehalococcoides* and *Dehalogenimonas* strains, the marine cluster I, and the marine cluster II. These results reveal that a higher ratio of horizontally transferred genetic materials was found in the *Dia* marine clusters compared to that of the *Dia* terrestrial cluster. Pangenome analysis further suggests that *Dia* microorganisms have evolved cluster-specific enzymes (e.g., dehalogenase in terrestrial *Dia*, sulfite reductase in marine *Dia*) and biosynthesis capabilities (e.g., siroheme biosynthesis in marine *Dia*). Marine *Dia* microorganisms are likely adapted to versatile metabolisms for energy conservation besides organohalide respiration. The genomic differences between marine and terrestrial *Dia* may suggest distinct functions and roles in element cycling (e.g., carbon, sulfur, chlorine), which require interdisciplinary approaches to unravel the physiology and evolution of *Dia* in various environments.

## Introduction

Members of the *Dehalococcoidia* (*Dia*) class microorganisms are frequently detected in various pristine and contaminated environments. Despite their widespread occurrence, the physiology, ecology, and evolution (e.g., nutritional requirements, niche adaption, metabolism traits) of *Dia* microorganisms remain largely unknown (Wasmund et al., 2016; Atashgahi et al., 2018; Schubert et al., 2018). While most studies had been focusing on *Dia* microorganisms as key players in the global biogeochemical cycling of organohalogens (Atashgahi et al., 2018), *in silico* analysis suggested underestimated *Dia* roles in carbon turnover (Kaster et al., 2014; Wasmund et al., 2014) and sulfur cycling (Wasmund et al., 2016) in natural environments (e.g., terrestrial, marine). Due to the limited *Dia* isolates obtained from pristine environments (e.g., marine sediments), difficulties in *Dia* cultivation and the lack of efficient genetic systems for heterologous expression of *Dia*-specific functional genes (e.g., reductive dehalogenase genes), characterization of *Dia* microorganisms from various environments had been partly relying on the bioinformatic analyses of metagenomes-assembled genomes (MAGs) and single-cell amplified genomes (SAGs) (Kaster et al., 2014; Wasmund et al., 2014; Fullerton and Moyer, 2016; Wasmund et al., 2016; Jungbluth et al., 2017; Parks et al., 2017; Sewell et al., 2017; Tully et al., 2018). For instance, 74, 36, and 5 draft-quality *Dia* MAGs were recovered from 1,500 metagenomes of different origins (Parks et al., 2017), 234 metagenomes from *Tara* Oceans Expedition (Tully et al., 2018), and 2 metagenomes from Juan de Fuca Ridge subseafloor fluid (Jungbluth et al., 2017), respectively. Despite the substantial increase in *Dia* genome information, an updated investigation of *Dia* microorganisms to unravel their biochemical, genetic, and evolutionary characteristics under close scrutiny has not been accomplished.

Based on 16S rRNA phylogeny, single-cell sequenced *Dia* cells (e.g., Dsc1, DscP2, DEH-J10) retrieved from marine sediments (e.g., Aarhus Bay, Peruvian Margin) are clustered within the previously termed ‘subphylum II’ clade of Chloroflexi phylum (Kaster et al., 2014; Wasmund et al., 2014; Sewell et al., 2017). These cells, prevalent in the marine subsurface, seemingly do not possess genes encoding for the reductive dehalogenase enzyme systems and are highly divergent from the cultivated representatives of organohalide respiring *Dia* (e.g., *Dehalococcoides* (*Dhc*), *Dehalogenimonas* (*Dhgm*), *Candidatus* “Dehalobium chlorocoercia”) (Adrian and Löffler, 2016). Thus, marine *Dia* microorganisms may utilize alternative strategies for energy conservation (e.g., substrate-level phosphorylation, dimethyl sulfoxide-DMSO-reduction) as encoded in their incomplete genomes. Reductive dehalogenase homologous (*rdh*) gene-like sequences were annotated in the single-cell sequenced DscP2 and *Dehalococcoidia* bacterium SCGC AB-540-C11 (i.e., DEH-C11) genomes, and further sequence analysis suggested the *rdh* genes in DscP2 and DEH-C11 genomes were dissimilar to the canonical *rdh* genes in *Dhc* and *Dhgm* and may encode for alternative functions (Kaster et al., 2014; Wasmund et al., 2016). Interestingly, *in silico* analysis indicated that all these single-cell sequenced microorganisms of marine origin cannot perform organohalide respiration, while all cultivated dehalogenating *Dia* microorganisms share a terrestrial origin (Kaster et al., 2014; Wasmund et al., 2014; Adrian and Löffler, 2016). Genomic and evolutionary comparisons between the marine and terrestrial *Dia* microorganisms hold promise to shed new insights into the physiology and evolution of *Dia* class.

The publicly available genomes archived in the JGI/IMG database (Joint Genome Institute, Integrated Microbial Genomes) offer a unique opportunity to explore the genomic features and evolution relationship between the terrestrial and marine *Dia* microorganisms. Given that previous studies mainly focused on a few single-cell sequenced *Dia* genomes, a generalized comparison of tens of single-cell and metagenome sequenced *Dia* genomes will generate more valuable information. Through the comparative genome analysis on single-cell and whole-genome sequenced *Dia* performed in this study, an improved understanding of the evolutionary relationship and insights into the key metabolic differences (e.g., organohalide respiration) among *Dia* microorganisms will be gained. These results expand our current understanding of *Dia* microorganisms regarding their evolutionary traits and functional diversity.

## Materials and Methods

### Phylogenetic Analysis

A total of 131 *Dia* genomes available in the JGI genome portal (genome.jgi.doe.gov) were selected for this study (Nordberg et al., 2014). RNAmmer v1.2 was used to retrieve the 16S rRNA genes from *Dia* genomes for the construction of phylogenetic trees (Lagesen et al., 2007). The 16S rRNA gene sequences of *Dhc* and *Dhgm* were retrieved from RDP (Ribosomal Database Project) database Release 11 Update 5 (Cole et al., 2014). By eliminating the genomes that did not possess a 16S rRNA gene with a minimum size of 1,000 bp, a total of 67 *Dia* genomes (Supplementary Table S1) were selected for downstream analysis. Due to the concern over the quality of metagenome-assembled genomes (Shaiber and Eren, 2019), we mainly focused on the whole-genome and single-cell sequenced *Dia* genomes. High-quality 16S rRNA gene sequences (>1,000 bp length) were compiled for phylogenetic inference analysis (Supplementary Dataset 1). Sequences were aligned with MUSCLE (version 3.8.31) using the UPGMB algorithm to build a guide tree for progressive alignment (Edgar, 2004), which was further curated with trimAI (version 1.4.1) with default settings (Capella-Gutierrez et al., 2009). The phylogenetic tree was built using a combined PhyML + SMS program (version 1.8.1) with SPR (Subtree Pruning and Regraphing) for searching tree topology and SH-like aLRT (Shimodaira–Hasegawa-like approximate Likelihood Ratio Test) for evaluating branch support (Guindon et al., 2010; Lefort et al., 2017; Lemoine et al., 2018). The construction of various 16S rRNA gene phylogenetic trees was completed using NGPhylogeny (Lemoine et al., 2019).

### Whole-Genome Comparison and *Dia* Pangenome

For genomic analysis and pangenome construction, *Dia* genomes were re-annotated using the RAST (Rapid Annotation using Subsystem Technology) tool with default parameters (Overbeek et al., 2014; Brettin et al., 2015) to ensure annotation conformity with formats and consistency across all genomes. All annotated coding sequences were verified by BLAST search (Altschul et al., 1990) against non-redundant protein sequences and by UniProt database search (e.g., UniProtKB reference proteomes plus Swiss-Prot) (The Uniprot Consortium, 2019). To minimize the effect of inconclusive information due to incomplete genomes, only genomes with sizes larger than 1.3 Mbp were selected for pangenome analysis; therefore, 14 genomes from the marine cluster I, 14 genomes from the marine cluster II, and 19 genomes from the terrestrial cluster were chosen for analysis (Supplementary Table S1). Pangenomes of different *Dia* clusters were constructed and analyzed by OrthoMCL (Li et al., 2003) with default parameters using computation capabilities provided by the KBase platform (Arkin et al., 2018). For the terrestrial cluster, the core protein families and core metabolic functions were defined if they are ubiquitously present in all analyzed genomes. The size of the completed genomes of the marine clusters was estimated to be about 2.36–2.84 Mbp based on the single-cell genomes DEH-J10 (1.44 Mbp with 60.8% completeness) (Wasmund et al., 2014) and DscP2 (1.38 Mbp with 85% completeness) (Kaster et al., 2014). For the marine clusters, the core protein families and the core metabolic functions were defined if they are present in 60% or more of the analyzed genomes. The Enzyme Commission numbers (EC numbers) of the core metabolic functions were retrieved and then analyzed with KEGG^1^ (Kanehisa, 2017; Kanehisa and Sato, 2020) and BRENDA^2^ (Jeske et al., 2019) to identify the key pathways and modules among the three *Dia* clusters. The statistical analysis was performed with JMP Pro version 13.2 (SAS Institute Inc., Cary, NC, United States).

### Molecular Clock Analysis

The divergence time for the *Dia* class from other classes of the Chloroflexi phylum was estimated by TimeTree (Kumar et al., 2017; Marin et al., 2017). Mega X (Kumar et al., 2018) for molecular evolutionary genetics analysis was applied to predict the divergence times of different *Dia* clusters by using the RelTime method (Tamura et al., 2012; Tamura et al., 2018) and the Tamura-Nei model (Tamura and Nei, 1993) following the established protocol (Mello, 2018). The 16S rRNA gene sequences were aligned with MUSCLE using the UPGMA algorithm and then analyzed for phylogeny reconstruction with the default maximum likelihood method using Mega X. The nucleotide sequences alignment and phylogenetic tree were used as the input for RelTime-ML. The divergence times of Chloroflexi (e.g., *Dhgm*) and Firmicutes (e.g., *Desulfitobacterium*) [2,925 to 3,379 Million Years Ago (MYA)] and of Chloroflexi (e.g., *Dhgm*) and Proteobacteria (e.g., *Anaeromyxobacter*) (2,263 to 3,551 MYA), estimated by TimeTree^3^ (Kumar et al., 2017; Marin et al., 2017), were applied to set the divergence time calibration constraints. The predicted divergence times were compared with those calculated by TimeTree for verification with a threshold of less than 20% difference following the published methods (McDonald and Currie, 2017).

### Re-analysis of Functional Coding Sequences

*Dia* functional genes (e.g., *rdh* gene, sulfite reductase gene, DMSO reductase gene) were retrieved from the IMG/MER database (img.jgi.doe.gov/cgi-bin/mer/main.cgi). All other sequences for building the phylogenetic inference tree were retrieved from Uniprot databases^4^ (The Uniprot Consortium, 2019). All protein sequences for building the phylogenetic trees of Rdhs were in the Supplementary Datasets 2. The phylogenetic tree for Rdhs was built by the “*a la carte*” pipeline of NGPhylogeny using the MUSCLE, trimAI, and PhyML + SMS with default settings (Lemoine et al., 2019).

### Analysis of Horizontal Gene Transfer Events

The horizontally transferred genes in each *Dia* genome were analyzed by the tool embedded in the JGI/IMG with default algorithms (Huntemann et al., 2015; Chen et al., 2017). Briefly, horizontally transferred genes are the genes that have their best BLAST hits (i.e., those with bit scores within 90% of the highest bit score) outside the taxonomic lineage of the genome (i.e., to genomes from another phylum, class). All the horizontally transferred genes were manually checked, and genes from the same taxonomic lineage were excluded. The correlation of the number of horizontally transferred genes to the total number of genes in each genome was analyzed following the previously described linear regression method (Jeong et al., 2016). Statistical analyses on the fitness of the linear relationship between the number of horizontally transferred genes and the total number of genes were performed by the JMP Pro version 13.2.

## Results

### The 16S rRNA Gene Phylogeny Separates *Dia* Microorganisms Into Distinct Terrestrial and Marine Clusters

**FIGURE 1:**
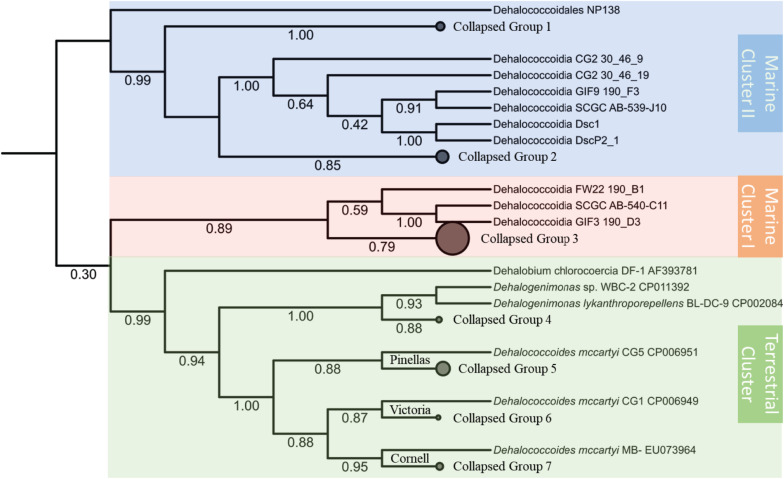
Phylogenetic inference tree of the 16S rRNA genes annotated from a total of 67 Dia genomes. The alignment of 16S rRNA genes were performed by MUSCLE and curated with trimAI. The phylogenetic tree was then built with the PhyML-SMS method. The Dia class of selected 67 genomes could be classified into three clusters: the terrestrial cluster (green area) including *Dhgm lykanthroporepellens* strain BL-DC-9 and *Dhc mccartyi* strains CG5, CG1 and MB; the marine cluster I (orange area) including Dehalococcoidia bacterium SCGC AB-540-C11; and the marine cluster II (blue area) including Dehalococcoidia bacterium SCGC AB-539-J10/DEH-J10. Some branches of the tree collapsed, and the genomes in each collapsed group are listed in the Supplementary Table S2. The branch support values of SH-like aLRT are labeled below each branch.

Despite the unresolved and insecure phylogenetic classification of Chloroflexi phylum (Gupta et al., 2013), the *Dia* class, separated from other classes of Chloroflexi, forms a cohesive cluster within the Chloroflexi phylum (Ravinesan and Gupta, 2014). The phylogenetic analysis based on the 16S rRNA gene sequences categorizes the *Dia* class into three clusters, the terrestrial cluster including all *Dhc* and *Dhgm* strains of terrestrial origin, the marine cluster I, and the marine cluster II (Figure 1). Such classification of *Dia* microorganisms into three clusters is consistent with the study of Chloroflexi phylogenetics for the Chloroflexi-related SAR202 bacterioplankton cluster genomes (Landry et al., 2017). The terrestrial cluster and the marine cluster I belong to previously classified subphylum IV of Chloroflexi, and the marine cluster II corresponds to the subphylum II of Chloroflexi (Hugenholtz et al., 1998; Inagaki et al., 2006; Wasmund et al., 2014). The separation of subphylum IV of Chloroflexi into two clusters (i.e., the terrestrial cluster and the marine cluster I) was also considered for their habitat-associated differences (i.e., marine versus terrestrial environments) that have likely emerged through evolutionary adaptation. Different alignment and phylogenetic tools were applied to evaluate *Dia* 16S rRNA phylogeny, and the topologies of these phylogenetic trees remain consistent and congruent with three major monophyletic clusters of *Dia*. High bootstrap values for each cluster (e.g., 0.99 for the terrestrial cluster, 0.89 for the marine cluster I, 0.99 for the marine cluster II) indicated the well-conserved monophyletic group for each cluster. Each *Dia* genome typically harbors a single copy of the 16S rRNA gene; by comparison, two copies of the 16S rRNA gene, only sharing an identity of 81.3%, were annotated on the DscP2 genome by RNAmmer. Our analysis indicated that these two 16S rRNA genes (IMG Gene ID 2584028172 and 2584028664) on the DscP2 genome were separated into the *Dia* marine cluster I and cluster II, respectively (Figure 1). This inconsistency was likely due to the artifact generated by the single-cell sequencing of DscP2 (e.g., wrong assembly) or the combination of two single-cell assembled genomes DscP2 and DscP3 (Kaster et al., 2014), which may introduce biased interpretation of *Dia* genomic characteristics and evolution of DscP2.

### Divergence Times Estimation for Different *Dia* Clusters

**FIGURE 2:**
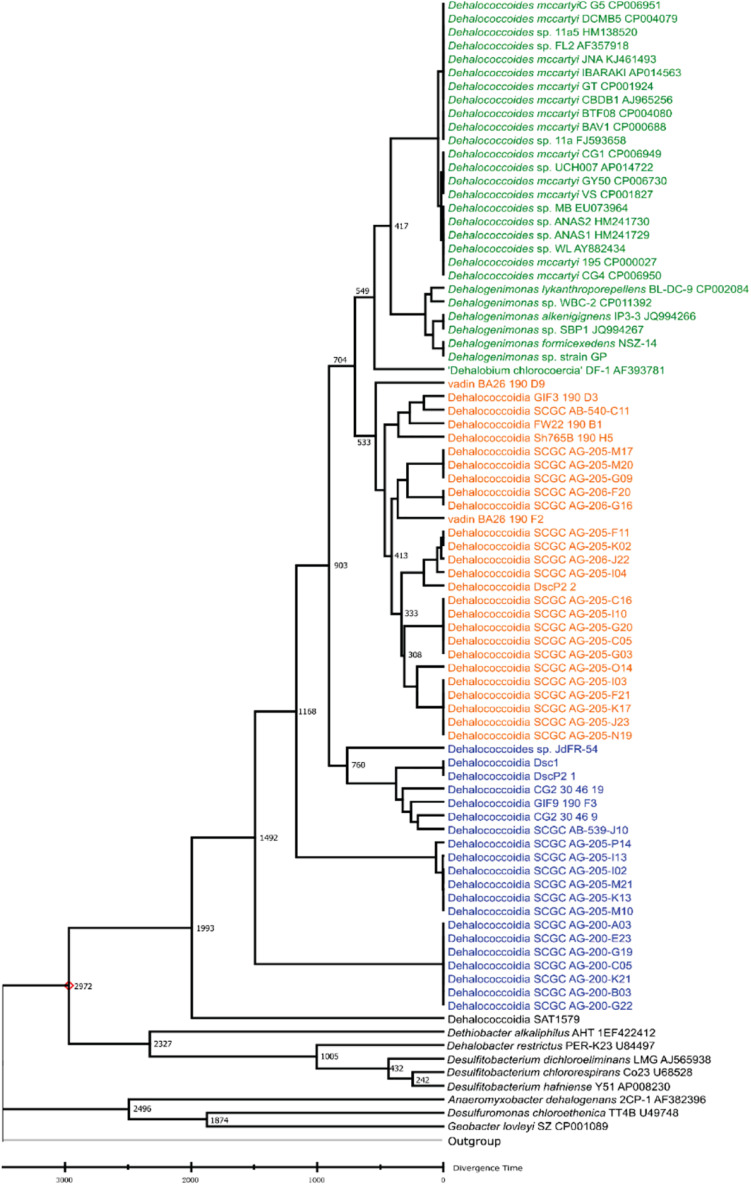
Phylogeny and molecular clock estimation of selected 16S rRNA genes from Dia class microorganisms and species from Firmicutes and Proteobacteria. *Acidobacterium capsulatum* ATCC 51196 is chosen as the outgroup. Branch lengths represent the divergence times (MYA), which were predicted and approximated by the Reltime method using Mega X. The terrestrial cluster, the marine cluster I and the marine cluster II are colored in green, orange and blue, respectively.

Various dynamic processes (e.g., mutation, horizontal gene transfer) affect the long-term evolution of *Dia* class microorganisms and the associated metabolisms (e.g., organohalide respiration), and the rates of these events are unknown through the geological time scale due to the lack of geological evidence (e.g., fossils) and biological evidence (e.g., biochemistry, genetics). We applied the RelTime approach to estimate divergent times among different *Dia* clusters with calibration constraints retrieved from TimeTree (Tamura et al., 2012; Kumar et al., 2017; Mello, 2018). The estimated logarithmic function of the likelihood value for time tree analysis is -21,719.56 involving 85 nucleotide sequences (i.e., a log-likelihood value of zero suggesting no support for the model, and the more negative indicates a good model fit). *Dia* class have diverged from other classes of Chloroflexi phylum approximately 1,791 MYA in the Paleoproterozoic era estimated by the TimeTree (Supplementary Figure S1). *Dhc* and *Dhgm* of the *Dia* terrestrial cluster and the marine cluster I were predicted to separate from the marine cluster II in the Neoproterozoic era (i.e., 1,000-541 MYA) (Figure 2), and the terrestrial cluster also diverged from the marine cluster I in the Neoproterozoic era. The TimeTree tool (see text footnote 3) estimated the divergence time for *Dhc* and *Dhgm* to have occurred in the Cambrian period (i.e., 541.0–485.4 MYA) (Supplementary Figure S1) compared with the Devonian period (i.e., 419.2–358 MYA) estimated by this study (Figure 2).

### Genome and Pangenome Comparison of Different *Dia* Clusters

The difference in average genome size of the terrestrial, the marine I and the marine II clusters was not statistically significant (ANOVA *p*-value = 0.0938) probably due to the incompleteness of most single-cell sequenced genomes. The available data could not support our hypothesis that the average genome size of the marine clusters was larger than that of the terrestrial cluster as a result of niche adaption (Martinez-Cano et al., 2014), and further investigation is required. For instance, the estimated genome sizes of DEH-J10 from the marine cluster II and DscP2 from the marine cluster I were relatively larger than those of the terrestrial Dia genomes.

To further explore the genomic differences among different *Dia* clusters, we performed a pangenome analysis to unravel the core functions and core protein families (i.e., shared by all genomes) in each *Dia* cluster using the OrthoMCL tool. The marine cluster I and cluster II have more singleton protein coding sequences and families than that of the terrestrial cluster, suggesting the genomes in marine clusters may have more unique functions not commonly shared and possibly could be sub-classified (Table 1). The ratios of the core functios against the total functions were in decreasing order for the marine cluster I, the terrestrial cluster and the marine cluster II, suggesting the core functions of *Dia* microorganimss of the marine cluster I were comparatively more conserved (Table 1).

**TABLE 1:** Pangenome statistics for different Dia clusters.

**Categories**		**Marine cluster I**	**Marine cluster II**	**Terrestrial cluster**
Total number of Genomes		14	14	19
Total number of protein CDs		26,620	27,093	30,310
	# of protein CDs in homolog families	23,148	22,972	27,648
	# of protein CDs singleton families	3,472	4,121	2,662
Total number of protein families		7,645	8,051	4,989
	# of homolog families	4,173	3,930	2,327
	# of singleton families	3,472	4,121	2,662
Number of Core families		941	822	812
Total number of functions		1,577	1,912	1,846
Number of Core functions		654	504	644

By comparing the core functions in each cluster (Supplementary Tables S4–S6), we found that certain functional genes are highly conserved in all *Dia* genomes (Supplementary Table S3). We further mapped core functional genes (i.e., EC numbers in Supplementary Table S3) against the BRENDA database, and found that the conserved genes were related to anabolism pathways (Supplementary Figure S3) (e.g., lipid A, isoprenoid, palmitate, arachidonate, lipid, aclacinomycin, *cis*-vaccenate, pantothenate, flavin, ppGpp), catabolism pathways (e.g., benzoyl-CoA, phenol, d-mannose, acetoin, propanol), central pathways of carbohydrate metabolisms (e.g., glycolysis, gluconeogenesis, citric acid cycle, pentose phosphate pathway), acetate fermentation, archaeal-type carbon dioxide fixation, tetrahydrofolate metabolism, sulfopterin metabolism, vitamin B_1_ metabolism, heme/siroheme metabolism, coenzyme A metabolism, purine and pyrimidine metabolisms, and amino acid metabolisms. For instance, genes coding for (R)-citramalate synthase, mainly present in archaeal genomes for the condensation of pyruvate and acetyl coenzyme A to 2-oxobutanoate (Howell et al., 1999), and 2-isopropylmalate synthase for the condensation of the acetyl group of acetyl-CoA with 3-methyl-2-oxobutanoate (2-oxoisovalerate) to form 3-carboxy-3-hydroxy-4-methylpentanoate (2-isopropylmalate), were present in genomes of three *Dia* clusters. Both enzymes are involved in the L-isoleucine biosynthesis.

In contrast, cluster-specific core functions, for example, core genes encoding for [Ni/Fe] hydrogenase, responsible for hydrogen utilization in *Dhc* and *Dhgm*, are present in all genomes of terrestrial Dia cluster, but not ubiquitously found in the genomes of marine *Dia* clusters. Genes encoding type I 3-dehydroquinate dehydratase, responsible for the production of aromatic acids in the shikimate pathway, is present in the genomes of the terrestrial cluster and marine cluster I but not the marine cluster II. The marine cluster II possesses the type II 3-dehydroquinate dehydratase gene, which participates in the shikimate pathway for phenylalanine, tyrosine and tryptophan biosynthesis for the conversion of 3-dehydroquinic acid to 3-dehydroshikimic acid. Gene coding for methylmalonyl-CoA mutase (EC 5.4.99.2), one of the most abundant cobamide-dependent enzymes in bacterial genomes (Sokolovskaya et al., 2019), were found in the genomes of *Dia* marine cluster I and II, but surprisingly, type II 3-dehydroquinate dehydratase and methylmalonyl-CoA mutase genes are missing in the genomes of the terrestrial cluster. N-methylhydantoinase (EC 3.5.2.14) gene, participating in arginine, creatinine, and proline metabolism by hydrolyzing N-methylimidazolidine-2,4-dione to N-carbamoylsarcosine is only present in the genomes of *Dia* marine clusters. Based on *Dia* genome annotation, the oxidation of complex fatty acids (e.g., 3-hydroxyacyl-CoA dehydrogenase, acetyl-CoA acetyltransferase) is a core function in the marine I and II clusters but not in the terrestrial cluster. Interestingly, these fatty acids oxidation genes were also found present in several *Dhgm* but not *Dhc* genomes. Likewise, genes encoding for heterodisulfide reductase (Hdr) subunits were present in the genomes of *Dia* marine clusters and *Dhgm* of the terrestrial cluster, but not in the *Dhc* genomes (Kaster et al., 2014; Wasmund et al., 2014; Yang et al., 2017).

### Putative *rdh* Genes in the Genomes of the Marine Cluster I Are Closely Related to Those Found in Non-*Dia* Microorganisms

**FIGURE 3:**
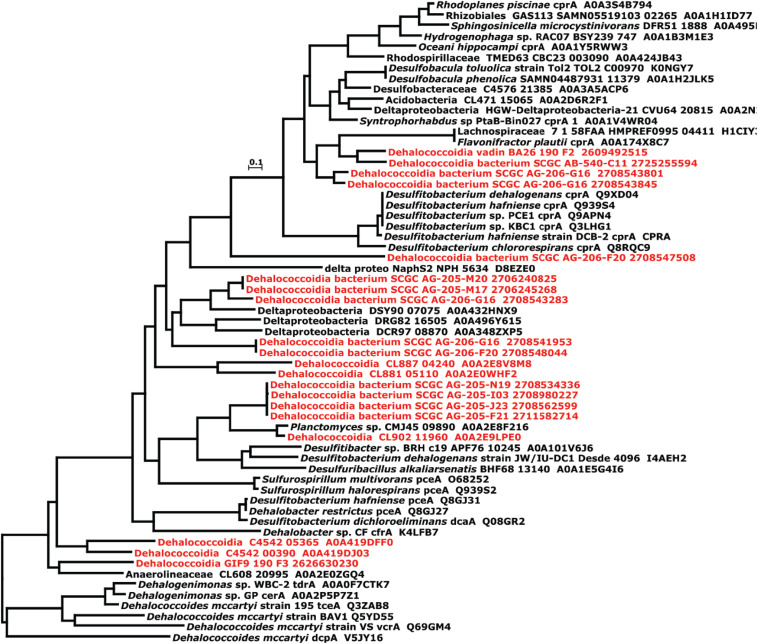
Phylogenetic tree of putative reductive dehalogenases annotated from the Dia marine clusters (Labeled in red). The phylogeny of putative reductive dehalogenases was constructed by the “a la carte” pipeline of ngphylogeny using the MUSCLE, trimAI, and PhyML + SMS in tandem with default settings. The scale bar represents the number of substitutions per site.

An important feature distinguishing the three *Dia* clusters is the presence of the *rdh* gene. All whole-genome sequenced *Dia* in the terrestrial cluster harbor multiple non-identical *rdh* genes (>5 coding sequences), while the genomes in the marine cluster I have none or only a few *rdh* genes (e.g., <5 coding sequences) (Supplementary Table S7). For example, one Rdh was annotated in the genome of DEH-C11 of the marine cluster I, and this Rdh was 32.3 and 34.9% similar to the non-respiratory cytoplasmic Rdh possessed by the aerobic marine bacteria *Comamonas* sp. strain 7D-2 and *Nitratireductor pacificus* strain pht3B, respectively (Wasmund et al., 2016). Two and four copies of *rdh* genes were annotated from the genomes of *Dehalococcoidia* bacterium SCGC AG-206-F20 and *Dehalococcoidia* bacterium SCGC AG-206-G16, respectively (Supplementary Table S7). Phylogenetic analysis suggested that the proteins encoded by the marine cluster I *rdh* genes share low sequence similarities (<20%) and do not cluster with those Rdh proteins from *Dhc* and *Dhgm* of the terrestrial cluster (Supplementary Figure S2) (Wasmund et al., 2016). The exact function of these *rdh* genes remains unknown, and could not be faithfully predicted by phylogenetic comparison since the closest related proteins are neither biochemically characterized nor functionally determined (Figure 3). In contrast, no *rdh* gene was identified in the genomes of *Dia* marine cluster II except for the *Dehalococcoidia* GIF9 190 F3 genome, which possesses a 696-bp coding sequence annotated as a partial *rdh* gene. The lack of biochemical evidence for the functions of these proteins hampered our understanding of the origins and evolutions of *rdh* genes. All examined *Dia* genomes in this study did not possess complete pathway for *de novo* biosynthesis of corrinoid (Supplementary Table S8), a requisite cofactor to complement the catalytic function of Rdhs. However, most genomes in the marine cluster I and the terrestrial cluster possess a largely incomplete set of genes (e.g., *cobA*, *cobU*, *cbiB*, *cobD*) (Yi et al., 2012) involved in corrinoid biosynthesis, salvage and remodeling (Supplementary Table S8), indicating that these *Dia* microorganisms must use the scavenging strategy to obtain exogenous corrinoid or its functional equivalent analogous to meet the need for corrinoid-dependent metabolisms (e.g., organohalide respiration).

### Siroheme-Dependent Respiration Metabolisms Are Associated With Marine *Dia*

Genes encoding for dissimilatory sulfite reductase and dimethyl sulfoxide (DMSO) reductase were identified in the genomes of *Dehalococcoidia* bacterium SCGC AB-540-C11 (Wasmund et al., 2016), *Dehalococcoidia* bacterium SCGC AG-205-M2, and *Dehalococcoidia* bacterium SCGC AG-205-I13 (Supplementary Tables S9, S10) of the *Dia* marine cluster II, and some genomes of the *Dia* marine cluster I (e.g., *Dehalococcoidia* bacterium SCGC AG-205-I10, *Dehalococcoidia* bacterium SCGC AG-205-M17, *Dehalococcoidia* bacterium SCGC AG-205-C05, *Dehalococcoidia* bacterium SCGC AG-205-G20) (Supplementary Tables S9, S10). In contrast, no dissimilatory sulfite reductase and DMSO reductase genes are generally found in the genomes of the Dia terrestrial cluster. Genes encoding for siroheme biosynthesis, which are required for the functional production of dissimilatory sulfite reductases, were also identified in the genomes of the marine clusters I, II, and two *Dehalogenimonas* genomes of the terrestrial cluster (Supplementary Table S8). These analyses suggested that siroheme-dependent respiration metabolisms (e.g., sulfite reduction) were not unique to some strains (e.g., DEH-C11), and could be a general electron transfer strategy for the marine *Dia* microorganisms. Instead, *Dhc* strains of the terrestrial cluster use a complex iron-sulfur molybdoenzyme (CISM) system (Kublik et al., 2016) for electron transport, which is consistent with the fact that *Dhc* strains do not possess the capability for siroheme biosynthesis (Schubert et al., 2018; Wang et al., 2018).

### Marine *Dia* Microorganisms Have a Higher Horizontal Gene Transfer Ratio Than That of the Terrestrial *Dia* Microorganisms

**FIGURE 4:**
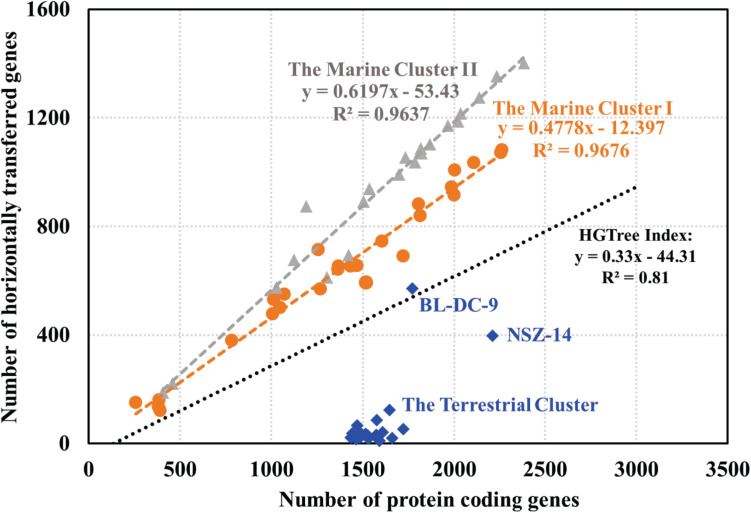
Relationship between the number of protein coding genes and number of predicted horizontally transferred genes. Each gray triangle, orange dot and blue square in the scatter-plot represents one microbial genome for the marine cluster II, the marine cluster I and the terrestrial cluster, respectively. The gray, orange and blue fitted regression lines describe the linear relationships between the number of predicted HGT-related genes and the total number of annotated genes for the Dia marine cluster II, the marine cluster I, and the terrestrial cluster, respectively. The black line, representing the linear relationship between the number of horizontally transferred genes and the total number of genes in each genome for a total of 2,472 prokaryotic genomes, is based on the previous study (Jeong et al., 2016).

Putative horizontally transferred genes were identified as their best BLAST hits outside the taxonomic lineage of the genome (Markowitz et al., 2010). Phage and transposase associated genes were found in the *Dia* genomes, suggesting the possible acquisition of genetic elements from other microbial groups. For instance, *rdh* genes within mobile genetic elements, were suggested to be recent horizontal acquisition (Adrian and Löffler, 2016). Analysis of the abundance of genetic exchange events among *Dia* genomes indicated differences in horizontal gene transfer ratio among the terrestrial and marine *Dia* clusters (Figure 4). A linear relationship exists between the total number of genes in each microbial genome and the total number of horizontal transfer genes as suggested by examining the HGTree database that is comprised of 2,472 completely sequenced prokaryotic genomes (*y* = −44.31 + 0.33X; *R*^2^ = 0.81) (Figure 4; Jeong et al., 2016). For the *Dia* marine clusters I and II, a higher percentage of horizontally transferred genes per genome than that of *Dia* terristial cluster was observed (Figure 4). The percentage ranges of horizontally transferred genes per marine *Dia* genome for the cluster I and cluster II were 31.12–58.37% and 46.44–73.59%, respectively. In contrast, a poor linear relationship between the total number of genes and the total number of horizontally transferred genes, as well as an overall lower abundance of horizontally transferred genes in the *Dia* terrestrial cluster genomes were found (Figure 4). Highest percentages of horizontally transferred genes were found in the genomes of *Dehalogenimonas lykanthroporepellens* strain BL-DC-9 (i.e., 32.24%) and *Dehalogenimonas formicexedens* strain NSZ-14 (i.e., 18.02%). Other than stain NSZ-14 and strain BL-DC-9, the average percentage of horizontal transferred genes in the *Dia* terrestrial cluster genomes was only 2.54% with a standard error 1.73%, which is much lower than the mean percentages of horizontal transferred genes in the *Dia* marine cluster I (i.e., 46.47%) and cluster II genomes (i.e., 57.74%) (*P* < 0.0001).

## Discussion

Microorganisms belonging to *Dhc* and *Dhgm* are known for their specialized energy metabolism utilizing organohalogens as electron acceptors, namely organohalide respiration. Whether other microorganisms belong to *Dia* class can also perform organohalide respiration is still uncertain due to the lack of representative cultured isolates. Tens of *Dia* microbial genomes assembled from metagenome or single-cell sequencing provide us an alternative approach to explore the physiology, biochemistry, ecology, and evolution of *Dia* microorganisms. By analyzing their genomic data, we identified the phylogenetic and genomic characteristics that separate the terrestrial and marine *Dia* microorganisms.

The phylum Chloroflexi is highly heterogeneous and includes a diversity of phenotypes and lifestyles. This heterogeneity is also apparent at the 16S rRNA gene phylogeny and genome levels, and a suggestion was made that the phylum Chloroflexi “*sensu stricto*” should only consist of the Chloroflexia and Thermomicrobia classes, while the other classes should be re-classified (Gupta et al., 2013). The same authors later suggested the placement of the *Dia* class within the Chloroflexi phylum was weakly supported based on the genomes of the terrestrial cluster (Ravinesan and Gupta, 2014). A recent study was proposed to standardize bacterial taxonomy based on genome phylogeny, and re-classified Chloroflexi phylum (“Chloroflexota”) into nine new classes (e.g., Anaerolineae, Chloroflexia, *Dia*, Ellin6529) (Parks et al., 2018). Despite unsettled phylogenetic positions of different phyla and classes due to an increased number of genome sequences (e.g., MAGs, SAGs), here we demonstrated that *Dia* microorganisms can be grouped into three clusters: the Dia terrestrial cluster, the *Dia* marine cluster I, and the Dia marine cluster II.

Based on the *in silico* genomic analysis, not all *Dia* class microorganisms (e.g., specifically, the marine cluster II) can perform reductive dehalogenation and are involved in the cycling of organohalides in the natural environment. For instance, the microorganisms of the *Dia* marine cluster II (e.g., *Dehalococcoidia* bacterium SCGC AB-539-J10 or DEH-J10) do not possess any *rdh* genes and are not expected to perform organohalide respiration, and their energy metabolism may depend on the oxidation of various fatty acids or other carbon substrates using beta-oxidation pathway (Wasmund et al., 2014). It has been suggested by other researchers that the *Dia* terrestrial cluster and marine cluster I may have acquired the *rdh* genes recently (Kaster et al., 2014). The ability of the marine cluster I *Dia* microorganisms to utilize halogenated substrates via reductive dehalogenation reaction needs to be experimentally verified. Therefore, the ecological roles of widespread *Dia* microorganisms, in terms of elements cycling, may be niche-dependent and cluster-specific. It was suggested that *Dia* should be specifically designated as those that can respire organohalide compounds, and those *Dia* unable to do so need be re-classified (Zinder, 2016), emphasizing the special role of *Dia* involved in global halogen cycle.

The Great Oxidation Event (i.e., 2400–2000 MYA) and Neoproterozoic Oxygenation Event (i.e., 800–500 MYA) are considered as two major steps in the oxygenation of the Earth’s surface (Supplementary Figure S1), which greatly affected the life and biological processes on Earth (Och and Shields-Zhou, 2012). For instance, the rise of oxygen was considered to have great impacts on altering iron mineral processes, one of the essential elements that affect the distribution of life on Earth, because of the increased difficulties of iron acquisition caused by the rise of oxygen levels (Kendall et al., 2012). By comparison, the isotopic signature found in the nitrate-assimilating organisms involved in aerobic nitrogen cycling indicated a widespread nitrate bioavailability due to the oxygenation (Kipp et al., 2018). Like the nitrogen cycle, the halogen cycle (e.g., halogenation and dehalogenation processes) is also potentially influenced by the oxgenated environments on early Earth. For example, halogenating enzymes (e.g., haloperoxidases, halogenases) requires oxygen to oxidize halogen elements and produce the organohalogens (Agarwal et al., 2017). It seems that the oxygenation events might play a role in the abiotic production of halogenated compounds (e.g., Fenton reaction, ferric iron) as well as increase and spread of halogenating enzymes, and thus, the promoted production of thousands of organohalogens on Earth. With the increased numbers of natural halogenated organic compounds, the evolution of dehalogenating microorganisms and the horizontal transfer rate of dehalogenating genes (e.g., haloacid dehalogenase genes, *rdh* genes) might be accelerated. The terrestrial Dia emerged approximately in the Cryogenian period of the Neoproterozoic era (i.e., 720–635 MYA) during the Neoproterozoic Oxygenation Event. Lack of information on the origins of organohalogens, the evolution of halogenating genes and dehalogenating genes have restricted our understandings of halogen cycling in the geological time scale. Biological approaches, including culture-dependent (e.g., enrichment, isolation, biochemistry, molecular genetics) and culture-independent methods (e.g., genomics, proteomics, metabolomics), combined with geochemistry have the potential to unravel the understudied co-evolution of *Dia* microorganisms and the halogen cycle both in the recent and distant past.

Why microorganisms of the *Dia* terrestrial cluster possess more *rdh* genes than those of the *Dia* marine clusters might be explained by two contradictory justifications. First, the diversity of halogenated organic substrates in the terrestrial environments induce the *Dia* microorganisms to produce a broader spectrum of *rdh* genes that allow for the utilization of various halogenated organic compounds. Another possible explanation is the terrestrial *Dia* microorganisms were forced to expand their tool inventories (e.g., *rdh*s) and scavenge the usable, but scarce halogenated organic compounds in the terrestrial environments. Further research is required to answer how *Dia* microorganisms and *rdh* genes evolve in the marine and terrestrial environments.

Heme and siroheme, the iron-porphyrin coordinated complexes, are the ubiquitously prosthetic groups that are involved in aerobic and anaerobic respiration, photosynthesis, reactive oxygen species, signaling and sensing of O_2_ and NO, and other redox reactions (e.g., nitrate reductase, nitrite reductases, sulfite reductases) (Hogle et al., 2014). Heme and siroheme biosynthesis pathway was believed to be an ancient metabolism with a long evolutionary history (Dailey et al., 2017). The absence of some genes for tetrapyrroles biosynthesis is common; however, the complete loss of genes encoding for *de novo* synthesis of tetrapyrrole molecules is uncommon except for *Dhc* and *Thermotoga* (Dailey et al., 2017). Dissimilatory sulfite reductase and DMSO reductase genes were identified in the *Dia* marine cluster I, and marine cluster II, indicating that *Dia* microorganisms were potentially involved in the global sulfur cycling. Despite the lack of experimental evidence for *Dia* capable of sulfite reduction, dissimilatory sulfite reductase genes were annotated in the genome of *Dehalococcoidia* bacterium SCGC AB-540-C11, indicating that the AB-540-C11 cell may be capable of sulfite or sulfate reduction. By comparison, no complete set of dissimilatory sulfite reductase genes was found in the genomes of *Dehalococcoidia* bacterium SCGC AG-205-M17 (marine cluster I), leading to the question as to what extent the *Dia* microorganisms are involved in the global sulfur cycling.

A higher ratio of genetic exchange and shuffling in the *Dia* marine cluster genomes than that in *Dia* terrestrial cluster genomes was predicted based on the algorithm developed by IMG/MER. No apparent explanations could be provided for such an observation since various factors (e.g., physical, biological, chemical parameters) may affect the frequency of horizontally transferred genes. For instance, stress conditions and SOS response, sub-inhibitory antimicrobials, dissemination of mobile genetic elements by plasmids and phages, and quorum sensing were possible mechanisms for the increased rates and numbers of horizontally transferred genes in different environmental microbiota (Aminov, 2011). The immense number of viruses and viral particles that populate marine environments have a tremendous effect on microbial diversity directly (e.g., selectively killing of microorganisms) and indirectly (e.g., genetic material cycling, horizontal gene transfer) (Suttle, 2007; Cai et al., 2019). Of note, the high virus abundance in ecosystems such as a wastewater treatment plant may also play a role in the acquisition of mobile genetic elements in the genome of *Dhc* strain 195 (Seshadri et al., 2005; Petrovich et al., 2019). Therefore, experimental evidence and *in silico* analyses are still required to unravel the mechanisms leading to comparatively more horizontally transferred genes in the *Dia* marine genomes.

In summary, the comparison of tens of assembled *Dia* genomes revealed the similarities and differences of evolutionary and genomic characteristics between the *Dia* terrestrial and marine clusters. A higher ratio of genetic exchange was found in the *Dia* marine clusters compared to the *Dia* terrestrial cluster. Additionally, the marine *Dia* microorganisms are likely to utilize different metabolisms for energy conservation rather than organohalide respiration. The differences between dehalogenating and non-dehalogenating *Dia* suggest that the two groups may play different ecological roles in the natural environment. The hypothesized evolutionary relationship between different clusters of the *Dia* class may reveal the evolution of the various enzymatic systems (e.g., dehalogenase, superoxide dismutase) in *Dia* microorganisms. Overall, the our analysis of these specialized *Dia* microorganisms shed light on the microbial evolution in the early Earth, as well as the role of organohalide-respiring microorganisms in the cycling of halogenated compounds, which aim to solicit advanced interdisciplinary research efforts to resolve the physiology and evolution of *Dia* microorganisms (e.g., more sampling and growth experiments, enrichment and cultivation, biochemical expression of targeted proteins, ecological and evolutionary modeling).

## Data Availability Statement

The datasets presented in this study can be found in online repositories. The names of the repository/repositories and accession number(s) can be found in the article/Supplementary Material.

## Author Contributions

YY and JY contributed to the design of the study, funding acquisition, supervision, and project administration. YZZ and YY performed the data curation and analysis. YY, NC, and JY wrote the first draft of the manuscript. All authors contributed to manuscript revision, read and approved the submitted version.

## Conflict of Interest

The authors declare that the research was conducted in the absence of any commercial or financial relationships that could be construed as a potential conflict of interest.

## Footnotes

1 https://www.kegg.jp

2 www.brenda-enzymes.org

3 www.timetree.org

4 www.uniprot.com

## Supplementary Material

FIGURE S1 Divergence times for selected microorganisms estimated and compiled by the TimeTree resource. The phylogenetic tree with precalculated divergence times and geochemical data were made and calculated by inputting a list of species name to the TimeTree tool. All dehalogenating microorganisms are labeled in red.

FIGURE S2 Phylogenetic tree of reductive dehalogenases. The putative reductive dehalogenases were annotated and retrieved from IMG/MER, and the details of these proteins could be found in the Supplementary Table S7. All the other reductive dehalogenases were retrieved from Uniprot databases. The phylogenic tree was built with the “a la carte” pipeline of ngphylogeny using the MUSCLE, trimAI, and PhyML + SMS with default settings. The putative reductive dehalogenases belonging to the marine clusters I and II are highlighted in yellow.

FIGURE S3 Main pathways of core funcitonal genes identified in the BRENDA by mapping the EC numbers in the in the Supplementary Table S3 to the BRENDA database.

TABLE S1 Details of Dia genomes utilized in this study. All information was compiled and retrieved from IMG/MER platform. The terrestrial cluster, marine cluster I and marine cluster II of selected microorganisms are shaded in light blue, light green, and light orange, respectively.

TABLE S2 List of Dia genomes for each collapsed group in the Figure 1. Each column represents a collapsed group. All collapsed group names were highlighted in bold.

TABLE S3 Analysis of core functions shared or uniquely possessed by the three clusters of Dia microorganisms (i.e., the terrestrial, marine I, marine II clusters). The numbers “0” and “1” indicate the absence and presence of the fucntion in each Dia cluster, respectively.

TABLE S4 Pangenome analysis of the marine cluster I including fourteen genomes with genome sizes larger than 1.3 Mbp in Supplementary Table S1.

TABLE S5 Pangenome analysis of the marine cluster II including fourteen genomes with genome sizes larger than 1.3 Mbp in Supplementary Table S1.

TABLE S6 Pangenome analysis of the terrestrial cluster including all nineteen genomes in Supplementary Table S1.

TABLE S7 List of putative reductive dehalogenases annotated from genomes of the marine cluster I and II.

TABLE S8 Comparison of genes encoding for the porphyrin and chlorophyll metabolism for the three clusters of Dia genomes using the KEGG map tool integrated within the IMG/MER platform with default parameters. The colored cells indicate the presence of genes while the empty cells indicate the absence of genes. The terrestrial cluster, marine cluster I and marine cluster II of selected microorganisms are shaded in light blue, light green, and light orange, respectively.

TABLE S9 List of dissimilatory sulfite reductase annotated from the three clusters of Dia genomes.

TABLE S10 List of DMSO reductases annotated from the three clusters of Dia genomes.
