# Supplementary figures and images for: Genomic Characteristics Distinguish Geographically Distributed Dehalococcoidia
Source: Front Microbiol. 2020 Sep 8;11:546063. doi: 10.3389/fmicb.2020.546063 (PMC7506110; doi:10.3389/fmicb.2020.546063)

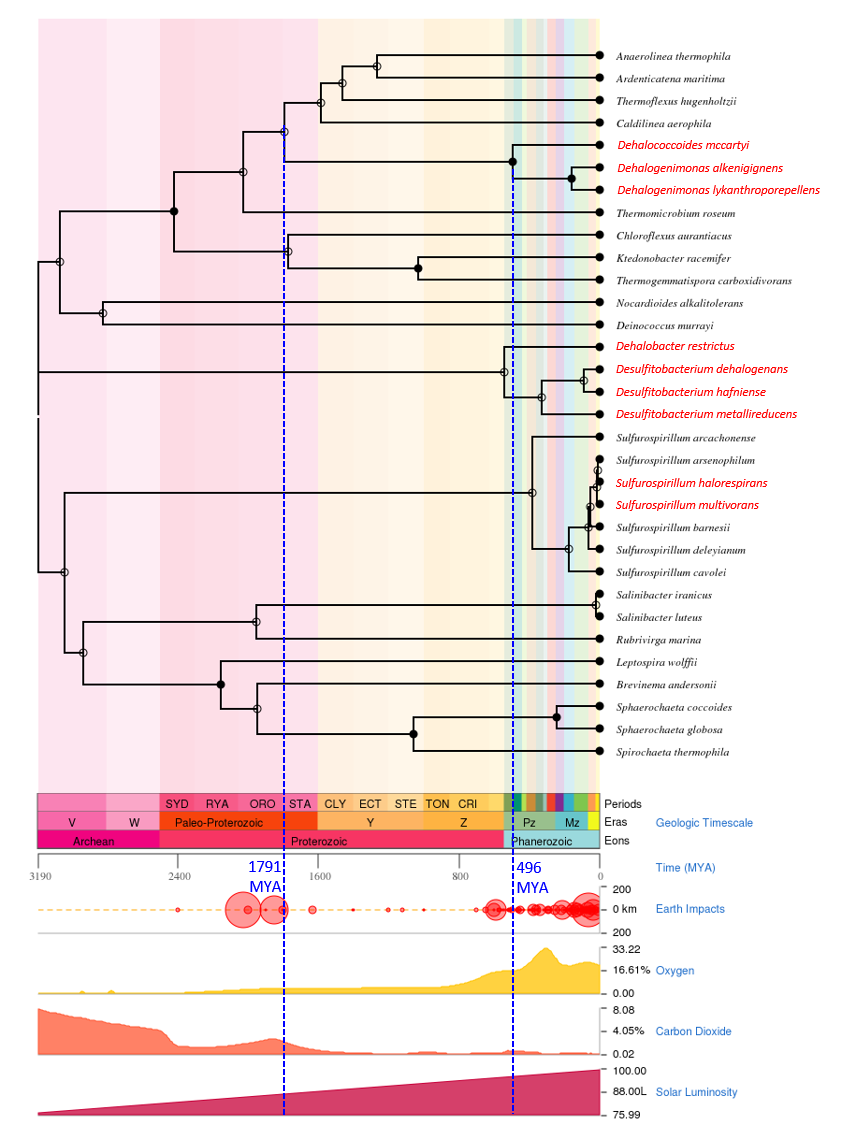

Supplement: FIGURE S1 — Divergence times for selected microorganisms estimated and compiled by the TimeTree resource. The phylogenetic tree with precalculated divergence times and geochemical data were made and calculated by inputting a list of species name to the TimeTree tool. All dehalogenating microorganisms are labeled in red. [file Image_1.TIF]

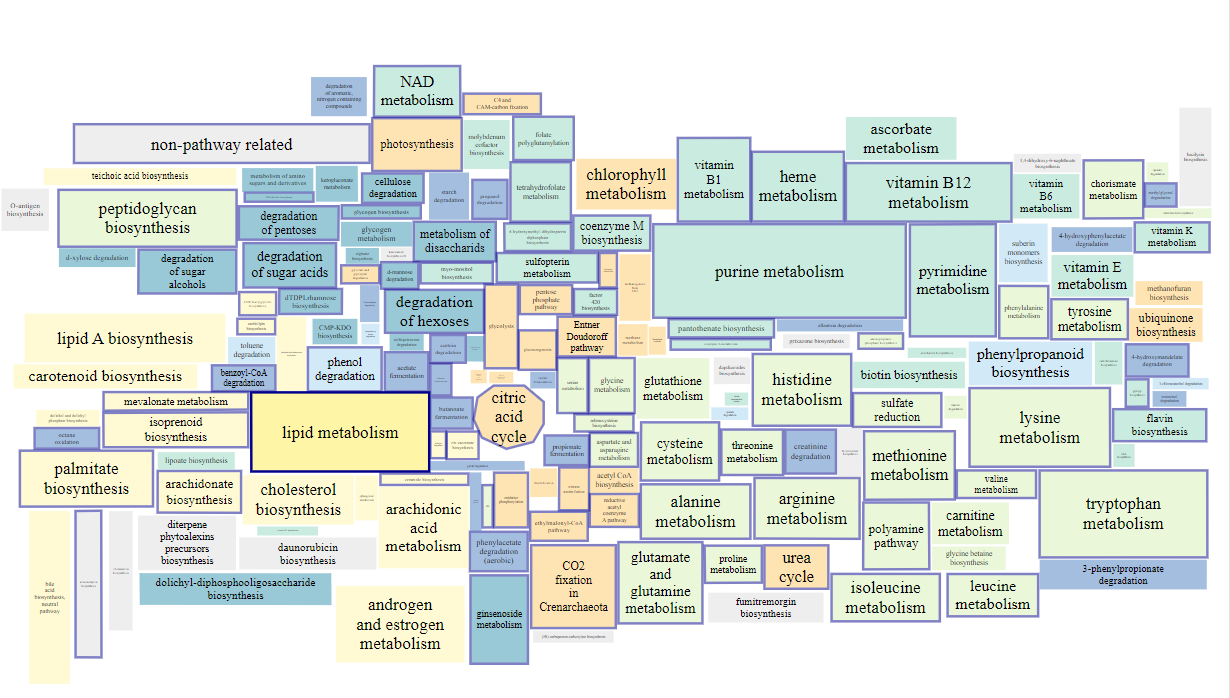

Supplement: FIGURE S3 — Main pathways of core funcitonal genes identified in the BRENDA by mapping the EC numbers in the in the Supplementary Table S3 to the BRENDA database. [file Image_3.PNG]
