# Supplementary material for: Genomic Characteristics Distinguish Geographically Distributed Dehalococcoidia
Source: Front Microbiol. 2020 Sep 8;11:546063. doi: 10.3389/fmicb.2020.546063 (PMC7506110; doi:10.3389/fmicb.2020.546063)
Supplement: FIGURE S2 — Phylogenetic tree of reductive dehalogenases. The putative reductive dehalogenases were annotated and retrieved from IMG/MER, and the details of these proteins could be found in the Supplementary Table S7. All the other reductive dehalogenases were retrieved from Uniprot databases. The phylogenic tree was built with the “a la carte” pipeline of ngphylogeny using the MUSCLE, trimAI, and PhyML + SMS with default settings. The putative reductive dehalogenases belonging to the marine clusters I and II are highlighted in yellow. [file Image_2.PDF]

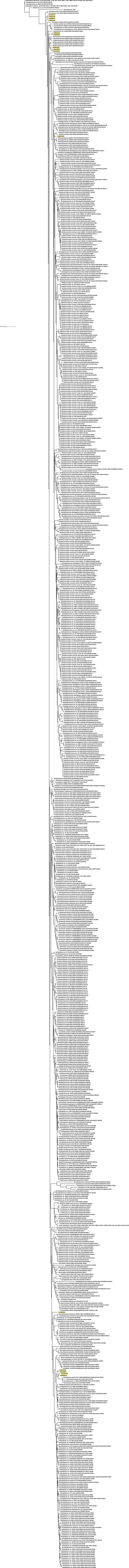
Sphingobium japonicum -strain DSM 16413 / CCM 7287 / MTCC 6362 / UT26 / NBRC 101211 / UT26S- linD LIND SPHJU
Sphingomonas sp- HZ-1 linD C9ELM1 9SPHN
Sphingomonas sp- NM05 linD Q0PV38 9SPHN
Sphingobium indicum -strain DSM 16412 / CCM 7286 / MTCC 6364 / B90A- linD LIND SPHIB
Rhodococcus sp- dmA A0A3G4BPC0 RHOSO
Clostridium sp- CAG
Dehalococcoidia CL902 11960 A0A2E9LPE0 9CHLR
Planctomyces sp- CMJ45 09890 A0A2E8F216 9PLAN
2711582714
2708562599
2708980227
2708534336
Nitrospinae CMH76 01390 A0A2D5UAL0 9BACT
Bacillus azotoformans MEV2011 M670 01440 A0A072NN08 BACAZ
Desulfuribacillus alkaliarsenatis BHF68 13140 A0A1E5G4I6 9BACL
Desulfitibacter sp- BRH c19 APF76 10245 A0A101V6J6 9FIRM
Desulfitobacterium dehalogenans strain JW/IU-DC1 Desde 4096 I4AEH2 DESDJ
Desulfarculus sp- C4525 00245 A0A3A6P803 9DELT
2708541953
2708548044
Actinobacteria ADurb-Bin444 cprA A0A1V5LGI9 9ACTN
Desulfobacteraceae EHM45 19495 A0A3N5QL42 9DELT
Deltaproteobacteria DRH11 03705 A0A496YVW2 9DELT
Deltaproteobacteria DCR97 08870 A0A348ZXP5 9DELT
Deltaproteobacteria DRG82 16505 A0A496Y615 9DELT
Deltaproteobacteria DSY90 07075 A0A432HNX9 9DELT
2708543283
2706240825
2706245268
Clostridiales DHW14 03680 A0A3D2KMH9 9FIRM
Clostridiales DGR79 04455 A0A3D5MN21 9FIRM
Clostridiales DHW14 08345 A0A3D2KSY6 9FIRM
Desulfitobacterium dehalogenans strain JW/IU-DC1 Desde 0041 I4A3K2 DESDJ
Desulfitobacterium sp- KBC1 prdA Q3LHG0 9FIRM
Dehalogenimonas lykanthroporepellens strain BL-DC-9 Dehly 1514 D8K2G9 DEHLB
Desulfitobacterium hafniense OX=49338 CPLR DESHA
Desulfitobacterium hafniense strain Y51 DSY1155 Q24YE8 DESHY
Spirochaetes ADurb-BinA120 cprA 1 A0A1V5HYA2 9SPIR
Dehalococcoides sp- MB OX=477183 C3RWI9 9CHLR
Dehalococcoides mccartyi rdhA A0A142V8E3 9CHLR
Dehalococcoides mccartyi strain 195 DET0311 Q3Z9P0 DEHM1
Dehalococcoides mccartyi CVH13 00864 A0A2J1DXP0 9CHLR
Dehalogenimonas formicexedens Dform 01462 A0A1P8F8I7 9CHLR
Dehalococcoides mccartyi C1G86 0948 A0A328ET66 9CHLR
Dehalococcoides mccartyi C1G87 1523 A0A328EJI2 9CHLR
Dehalococcoides mccartyi C1O63 0470 A0A2S5D8R9 9CHLR
Dehalogenimonas formicexedens dcpA A0A1P8F8L0 9CHLR
Dehalogenimonas lykanthroporepellens strain BL-DC-9 Dehly 1524 D8K2H9 DEHLB
Dehalogenimonas alkenigignens DEALK 17200 A0A0W0GJX3 9CHLR
Dehalococcoides mccartyi dcpA V5JY16 9CHLR
Dehalococcoides mccartyi C1G87 1524 A0A328ELI9 9CHLR
Dehalococcoides mccartyi dcpA V5JY56 9CHLR
Dehalogenimonas alkenigignens DEALK 00330 A0A0W0GKP1 9CHLR
Dehalogenimonas formicexedens Dform 02003 A0A1P8FA25 9CHLR
Dehalococcoides mccartyi rdhA14 A0A059U7V1 9CHLR
Dehalococcoides mccartyi C1G86 0258 A0A328ESP9 9CHLR
Dehalococcoides mccartyi rdhA23 A0A0A7NUV9 9CHLR
Dehalococcoides mccartyi rdhA A0A142V8B0 9CHLR
Dehalococcoides mccartyi strain 195 DET0235 Q3Z9W6 DEHM1
Dehalococcoides mccartyi DA01 08075 A0A0V8LXD7 9CHLR
Dehalococcoides sp- MB mbrA D6MZ08 9CHLR
Dehalococcoides mccartyi CVH13 01251 A0A2J1DVE6 9CHLR
Dehalogenimonas sp- GP JP09 005870 A0A2P5P7Q3 9CHLR
Dehalococcoides mccartyi rdhA11 A0A059U475 9CHLR
Dehalococcoides mccartyi rdhA A0A142VDZ4 9CHLR
Dehalococcoides mccartyi B1775 00335 A0A1S7ARW0 9CHLR
Dehalococcoides mccartyi strain 195 DET1559 Q3Z693 DEHM1
Dehalogenimonas sp- WBC-2 DGWBC 0133 A0A0F7GCK0 9CHLR
Dehalogenimonas alkenigignens DEALK 06060 A0A0W0GGU2 9CHLR
Dehalogenimonas lykanthroporepellens strain BL-DC-9 Dehly 0849 D8K0M3 DEHLB
Dehalogenimonas lykanthroporepellens strain BL-DC-9 Dehly 0068 D8K383 DEHLB
Dehalogenimonas alkenigignens DEALK 16100 A0A0W0GJM1 9CHLR
Dehalogenimonas formicexedens Dform 01364 A0A1P8F8B0 9CHLR
Dehalococcoidia C4542 00390 A0A419DJ03 9CHLR
Dehalococcoidia C4542 05365 A0A419DFF0 9CHLR
2626630230
Rhodospirillaceae CMM10 12785 A0A2D6G2J4 9PROT
Rhodospirillaceae CMM10 07605 A0A2D6FZK0 9PROT
Rhodospirillaceae CMM74 07815 A0A2E7QFT3 9PROT
Anaerolineaceae CL607 21965 A0A2E1AIK2 9CHLR
Anaerolineaceae CL607 21975 A0A2E1AIJ6 9CHLR
Anaerolineaceae CL608 20995 A0A2E0ZGQ4 9CHLR
Desulfitibacter sp- BRH c19 APF76 10275 A0A101V6N0 9FIRM
Desulfitobacterium dehalogenans strain JW/IU-DC1 Desde 4095 I4AEH1 DESDJ
Desulfoluna spongiiphila SAMN05216233 102299 A0A1G5C0H8 9DELT
Lutibacter oricola SAMN05444411 102301 A0A1H2WW13 9FLAO
Marinifilaceae SPP2 ALGA 1590 A0A1Y1CHW0 9BACT
Maribacter polysiphoniae LX92 03527 A0A316DTX0 9FLAO
Dehalococcoides mccartyi CVH13 00192 A0A2J1E078 9CHLR
Dehalococcoides mccartyi -strain VS- rdhA D2BG05 DEHMV
Dehalococcoides mccartyi CVH13 01544 A0A2J1DT91 9CHLR
Dehalogenimonas formicexedens Dform 00370 A0A1P8F5M3 9CHLR
Dehalogenimonas sp- GP JP09 002065 A0A2P5P8Q8 9CHLR
Dehalogenimonas lykanthroporepellens strain BL-DC-9 Dehly 1355 D8K216 DEHLB
Dehalogenimonas sp- WBC-2 DGWBC 1640 A0A0F7GFX3 9CHLR
Dehalococcoides mccartyi CVH13 01062 A0A2J1DWK1 9CHLR
Dehalococcoides mccartyi CVH13 00076 A0A2J1E0J0 9CHLR
Dehalococcoides mccartyi strain BAV1 RdhA3 Q673E0 DEHMB
Dehalococcoides mccartyi CVH13 00095 A0A2J1E0J9 9CHLR
Dehalogenimonas lykanthroporepellens strain BL-DC-9 Dehly 0283 D8K3T7 DEHLB
Dehalococcoides mccartyi DA01 00205 A0A0V8M582 9CHLR
Dehalococcoides mccartyi C1G87 1448 A0A328EKB3 9CHLR
Dehalococcoides mccartyi C1G86 1483 A0A328ENC2 9CHLR
Dehalococcoides mccartyi rdhA19 A0A059U7W2 9CHLR
Dehalogenimonas lykanthroporepellens strain BL-DC-9 Dehly 1148 D8K1G6 DEHLB
Dehalococcoides mccartyi C1G86 1487 A0A328ENQ0 9CHLR
Dehalococcoides mccartyi strain 195 DET1528 Q3Z6C3 DEHM1
Dehalococcoides mccartyi OX=61435 Q67B55 9CHLR
Dehalococcoides mccartyi pteA A0A142VC48 9CHLR
Dehalococcoides mccartyi -strain VS- rdhA D2BJC8 DEHMV
Dehalococcoides mccartyi OX=61435 Q67B47 9CHLR
